# Variational principle for scale-free network motifs

**DOI:** 10.1038/s41598-019-43050-8

**Published:** 2019-05-01

**Authors:** Clara Stegehuis, Remco van der Hofstad, Johan S. H. van Leeuwaarden

**Affiliations:** Department of Mathematics and Computer Science, Eindhoven University of Technology, Eindhoven, Netherlands

**Keywords:** Applied mathematics, Complex networks

## Abstract

For scale-free networks with degrees following a power law with an exponent *τ* ∈ (2, 3), the structures of motifs (small subgraphs) are not yet well understood. We introduce a method designed to identify the dominant structure of any given motif as the solution of an optimization problem. The unique optimizer describes the degrees of the vertices that together span the most likely motif, resulting in explicit asymptotic formulas for the motif count and its fluctuations. We then classify all motifs into two categories: motifs with small and large fluctuations.

## Introduction

Many real-world networks, like communication networks, social networks and biological networks, were found to be *scale free* with power-law degree distributions and infinite variance in the large-network limit^1–5^. The heavy tail of the power law comes with *hubs*, vertices of extremely high degree. Hubs create ultra-small distances, ultra-fast information spreading and resilience against random attacks, while the average node degree is small. Attested by real-world data and explained by mathematical models, the consequences of power-law connectivity patterns now belong to the foundations of network science.

Scale-free networks can be studied using random network models that connect vertices through edges forming power-law degree distributions. Connectivity patterns beyond edges are commonly described in terms of small subgraphs called motifs (or graphlets). There is increasing interest in algorithmic methods to count motifs^6–10^, or the relation between motifs and network functions, like the spread of epidemics^11–16^. Motifs can describe the tendency for clustering and other forms of network organization^17–19^.

Much existing work focuses on the classification of complex networks in terms of motif counts or frequencies. The occurrence of specific motifs such as wedges, triangles and cliques have been proven important for understanding real-world networks. Indeed, motif counts might vary considerably across different networks^20–22^ and any given network has a set of statistically significant motifs. Statistical relevance can be expressed by comparing real-world networks to mathematically tractable models. A popular statistic takes the motif count, subtracts the expected number of motifs in the mathematical model, and divides by the standard deviation in the mathematical model^22–24^. Such a standardized test statistic predicts whether a motif is overrepresented in comparison to some mathematical model. This comparison, however, filters out the effect of the degree sequence, the network size and possibly other features that are needed to understand the structure and frequency of motifs.

With the goal to explain the occurrence of motifs beyond counting, we develop a method to identify, for any given motif, the composition that dominates the motif count as the solution of an optimization problem. The unique optimizer describes the degrees of the vertices that together span the most likely motif, as well as predicts the leading asymptotic order for the motif count in the large-network limit. Our method can potentially be applied to various random network models, but is developed first for the hidden-variable model^25–31^, a random network model that generates graphs with power-law degrees. Given *n* vertices, the hidden-variable model associates to each node a hidden variable *h* drawn independently from the probability density

1 $$\rho (h)=C{h}^{-\tau }$$

for some constant *C* and $h\ge {h}_{{\rm{\min }}}$. Next, conditionally on all the hidden variables, each pair of vertices is joined independently with probability

2 $$p(h,h^{\prime} )=\,{\rm{\min }}(hh^{\prime} /(\mu n),1).$$

with *h* and *h*′ the hidden variables associated with the two vertices, and *μ* the mean of the hidden variables. For any given motif, we now seek for its most likely structure. The probability *P*(*H*) of creating motif *H* on *k* uniformly chosen vertices can be written as

3 $$P(H)={\int }_{{\boldsymbol{h}}}\,{\mathbb{P}}\,(H\,{\rm{on}}\,{h}_{1},\ldots ,{h}_{k})\,{\mathbb{P}}\,({h}_{1},\ldots ,{h}_{k})\,{\rm{d}}{\boldsymbol{h}},$$

where the integral is over all possible hidden-variable sequences on *k* vertices, with ${\boldsymbol{h}}=({h}_{1},\ldots ,{h}_{k})$ and ${\mathbb{P}}\,({h}_{1},\ldots ,{h}_{k})$ the density that a randomly chosen set of *k* hidden variables is proportional to ${h}_{1},\ldots ,{h}_{k}$. The degree of a node is asymptotically Poisson distributed with its hidden variable as mean^32^, so (3) can be interpreted as a sum over all possible degree sequences. Therefore, our optimization method then needs to settle the following trade-off, inherently present in power-law networks: On the one hand, large-degree vertices contribute substantially to the number of motifs, because they are highly connected, and therefore participate in many motifs. On the other hand, large-degree vertices are by definition rare. This should be contrasted with lower-degree vertices that occur more frequently, but take part in fewer connections and hence fewer motifs. Therefore, our method give rise to a certain ‘variational principle’, because it finds the selection of vertices with specific degrees that together ‘optimize’ this trade-off and hence maximize the expected number of such motifs.

We leverage the optimization method in two ways. First, we derive sharp expressions for the motif counts in the large-network limit in terms of the network size and the power-law exponent. Second, we use the method to identify the fluctuations of motif counts.

We present two versions of the method that we call free and typical variation. Free variation corresponds to computing the average number of motifs over many samples of the random network model. Typical variation corresponds to the number of motifs in one single instance of the random graph model. Remarkably, for $\tau \in (2,3)$ these can be rather different. After that, we apply the method to study motif count fluctuations. Finally, we provide a case study where we investigate the presence of motifs in some real-world network data.

## Results

### Free variation

We first show that only hidden-variable sequences ***h*** with hidden variables of specific orders give the largest contribution to (3). Write the hidden variables as ${h}_{i}\propto {n}^{{\alpha }_{i}}$ for some ${\alpha }_{i}\ge 0$ for all *i*. Then, using (2), the probability that motif *H* exists on vertices with hidden variables ${\boldsymbol{h}}=({n}^{{\alpha }_{1}},\ldots ,{n}^{{\alpha }_{k}})$ satisfies

4 $${\mathbb{P}}\,(H\,{\rm{on}}\,{\boldsymbol{h}})\propto \prod _{({v}_{i},{v}_{j})\in {E}_{H}:{\alpha }_{i}+{\alpha }_{j} < 1}\,{n}^{{\alpha }_{i}+{\alpha }_{j}-1}.$$

The hidden variables are an i.i.d. sample from a power-law distribution, so that the probability that *k* uniformly chosen hidden variables satisfy $({h}_{1},\ldots ,{h}_{k})\propto ({n}^{{\alpha }_{1}},\ldots ,{n}^{{\alpha }_{k}})$ is of the order ${n}^{(1-\tau ){\sum }_{i}{\alpha }_{i}}$ (see Supplementary Material 2). Taking the product of this with (4) shows that the maximum contribution to the summand in (3) is obtained for those ${\alpha }_{i}\ge 0$ that maximize the exponent

5 $$(1-\tau )\,\sum _{i}\,{\alpha }_{i}+\sum _{(i,j)\in {E}_{H}:{\alpha }_{i}+{\alpha }_{j} < 1}\,({\alpha }_{i}+{\alpha }_{j}-1),$$

which is a piecewise-linear function in *α*. In Supplementary Material 2, we show that the maximizer of this optimization problem satisfies ${\alpha }_{i}\in \{0,\tfrac{1}{2},1\}$ for all *i*. Thus, the maximal value of (5) is attained by partitioning the vertices of *H* into the sets *S*_1_, *S*_2_, *S*_3_ such that vertices in *S*_1_ have ${\alpha }_{i}=0$, vertices in *S*_2_ have ${\alpha }_{i}=1$ and vertices in *S*_3_ have ${\alpha }_{i}=\tfrac{1}{2}$. Then, the edges with ${\alpha }_{i}+{\alpha }_{j} < 1$ are edges inside *S*_1_ and edges between *S*_1_ and *S*_3_. If we denote the number of edges inside *S*_1_ by ${E}_{{S}_{1}}$ and the number of edges between *S*_1_ and *S*_3_ by ${E}_{{S}_{1},{S}_{3}}$, then maximizing (5) is equivalent to maximizing

6 $${B}_{f}(H)=\mathop{{\rm{\max }}}\limits_{{\mathscr{P}}}\,[|{S}_{1}|-|{S}_{2}|-\frac{2{E}_{{S}_{1}}+{E}_{{S}_{1},{S}_{3}}}{\tau -1}]$$

over all partitions ${\mathscr{P}}$ of the vertices of *H* into *S*_1_, *S*_2_, *S*_3_. This gives the following theorem (a more elaborate version is proven in Supplementary Material 2):

#### Theorem 1

(Expected motif count). *Let H be a motif on k vertices such that the solution to* (6) *is unique*. *As* $n\to \infty$, *the expected number of motifs H grows as*

7 $${\mathbb{E}}\,[N(H)]={n}^{k}P(H)\propto {n}^{\frac{3-\tau }{2}k+\frac{\tau -1}{2}{B}_{f}(H)},$$

*and is thus fully determined by the partition* ${{\mathscr{P}}}^{\ast }$ *that optimizes* (6).

**Figure 1:**
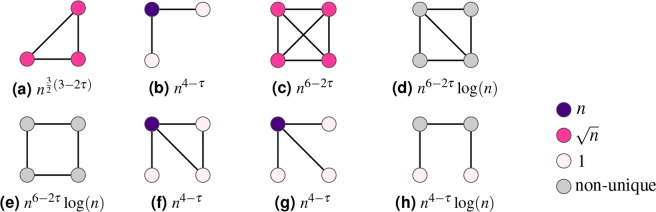
Scaling of the expected number of motifs on 3 and 4 vertices in *n*, where the vertex color indicates the dominant vertex degree. Vertices where the optimizer is not unique are gray.

**Figure 2:**
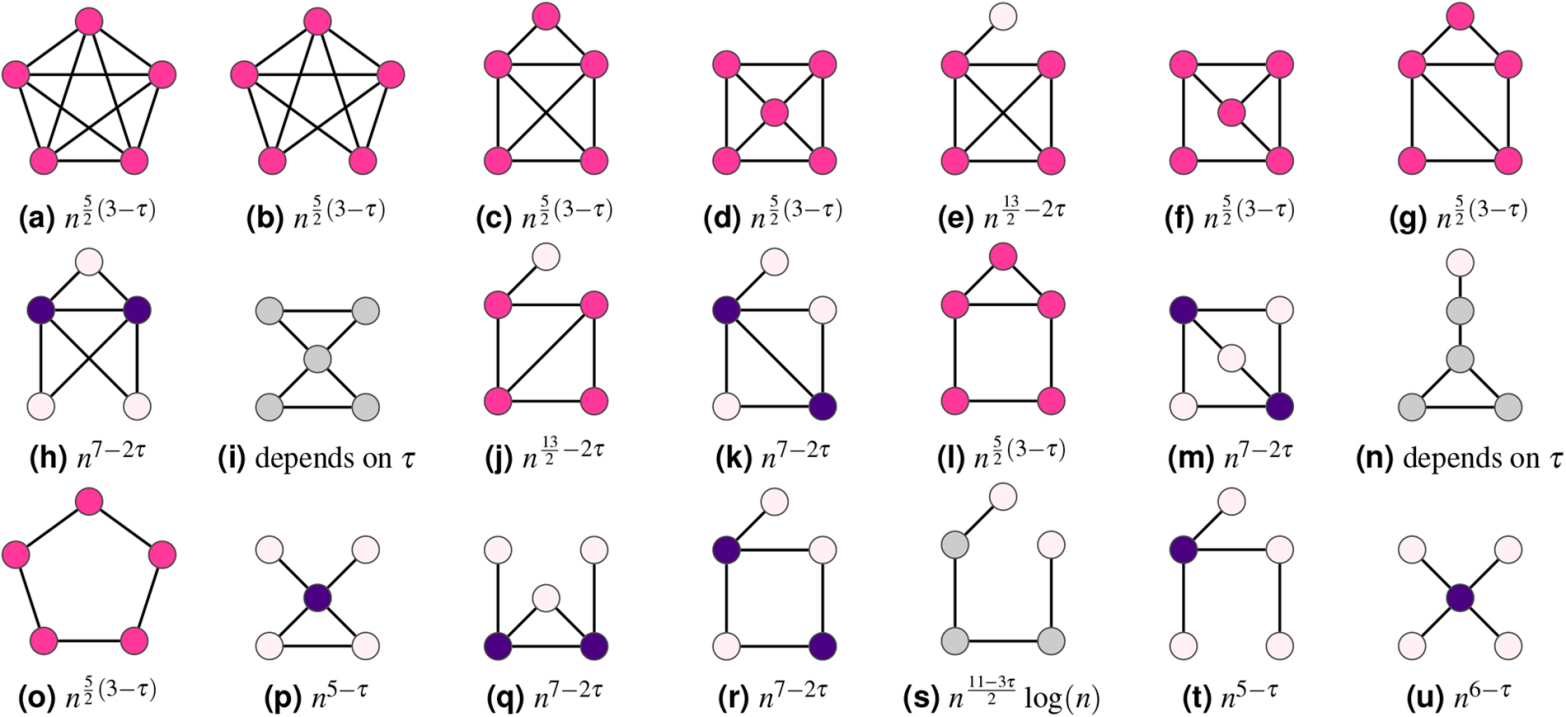
Scaling of the expected number of motifs on 5 vertices in *n*, where the vertex color indicates the dominant vertex degree, as in Fig. 1.

Theorem 1 implies that the expected number of motifs is dominated by motifs on vertices with hidden variables (and thus degrees) of specific orders of magnitude: constant degrees, degrees proportional to $\sqrt{n}$ or degrees proportional to *n*. Figures 1 and 2 show the partitions ${{\mathscr{P}}}^{\ast }$ that dominate the expected number of motifs on three, four and five vertices.

### Typical variation

The largest degrees (hubs) in typical samples of the hidden-variable model scale as ${n}^{1/(\tau -1)}$ with high probability. The expected number of motifs, however, may be dominated by network samples where the largest degree is proportional to *n* (see Theorem 1). These samples contain many motifs because of the high degrees, and therefore contribute significantly to the expectation. Nevertheless, the probability of observing such a network tends to zero as *n* grows large. We therefore now adapt the variational principle with the goal to characterize the typical motif structure and hence the typical number of motifs.

We again assume degrees to be proportional to ${n}^{{\alpha }_{i}}$, but now limit to degree sequences where the maximal degree is of order ${n}^{1/(\tau -1)}$, the natural cutoff in view of the typical hub degrees. The dominant typical motif structure is then obtained by maximizing (5), with the additional constraint that ${\alpha }_{i}\le \tfrac{1}{\tau -1}$. In Supplementary Material 3 we show that the possible optimizers are one of four values ${\alpha }_{i}\in \{0,\tfrac{\tau -2}{\tau -1},\tfrac{1}{2},\tfrac{1}{\tau -1}\}$, and obtain an optimization problem similar to (6).

**Figure 3:**
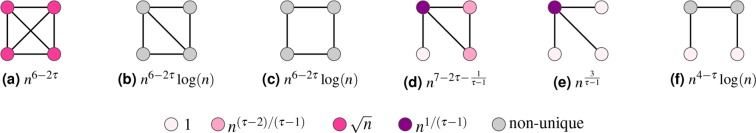
Typical scaling of the number of motifs on three or four vertices in *n*. The vertex color indicates the dominant vertex degree.

This shows that the typical degree of a motif is of constant order or proportional to ${n}^{1/(\tau -1)}$, $\sqrt{n}$ or ${n}^{(\tau -2)/(\tau -1)}$. Figure 3 and Supplementary Fig. 1 show the most likely motifs on three, four and five vertices. Observe that the dominant structures and the number of motifs of Figs 1 and 3 may differ. For example, the scaling of the expected number of claws (Fig. 1b) and the typical number of claws (Fig. 3b) is different. This is caused by the left upper vertex that has degree proportional to *n* in the free dominant structure, whereas its typical degree is proportional to ${n}^{1/(\tau -1)}$. Only when the solution to (6) does not involve hub vertices, the two scalings coincide. Hub vertices in the dominant structure give a major contribution to the motif count. While typical hub degrees scale as ${n}^{1/(\tau -1)}$, expected hub degrees may be much larger, causing the number of such motifs with hubs to scale faster in the free variation setting than in the typical variation setting. This indicates that the average and median motif count can differ dramatically.

#### Graphlets

It is also possible to only count the number of times *H* appears as an induced subgraph, also called graphlet counting. This means that an edge that is not present in graphlet *H*, should also be absent in the network motif. In Supplementary Material 4 we classify the expected and typical number of graphlets with a similar variational principle as for motifs. Supplementary Fig. 2 shows the typical behavior of graphlets on 4 vertices. This figure also shows that graphlet counting is more detailed than motif counting. For example, counting all square motifs is equivalent to counting all graphlets that contain the square as an induced subgraph: the square, the diamond and *K*_4_. Indeed, we obtain that the number of square motifs scales as ${n}^{6-2\tau }\,\mathrm{log}(n)$ by adding the number of square, diamond and *K*_4_ graphlets from Supplementary Fig. 2. This shows that most square motifs are actually the diamond graphlets of Supplementary Fig. 2b. Thus, graphlet counting gives more detailed information than motif counting.

### Fluctuations

Self-averaging network properties have relative fluctuations that tend to zero as the network size *n* tends to infinity. Several physical quantities in for example Ising models, fluid models and properties of the galaxy display non-self-averaging behavior^33–39^. We consider motif counts *N*(*H*) and call *N*(*H*) self-averaging when ${\rm{Var}}\,(N(H))/{\mathbb{E}}\,{[N(H)]}^{2}\to 0$ as $n\to \infty$. Essential understanding of *N*(*H*) can then be obtained by taking a large network sample, since the sample-to-sample fluctuations vanish in the large-network limit. In contrast, if ${\rm{Var}}\,(N(H))/{\mathbb{E}}\,{[N(H)]}^{2}$ approaches a constant or tends to infinity as $n\to \infty$, the motif count is called non-self-averaging, in which case *N*(*H*) shows (too) strong sample-to-sample fluctuations that cannot be mitigated by taking more network samples.

Our variational principle facilitates a systematic study of such fluctuations, and leads to a classification into self-averaging and non-self-averaging for all motifs *H*. It turns out that whether *N*(*H*) is self-averaging or not depends on the power-law exponent $\tau$ and the dominant structure of *H*. We also show that non-self-averaging behavior of motif counts may not have the intuitive explanation described above. In some cases, motif counts in two instances are similar with high probability, but rare network samples behave differently, causing the motif count to be non-self-averaging. Thus, the classification of network motifs into self-averaging and non-self-averaging motifs does not give a complete picture of the motif count fluctuations. We therefore further divide the non-self-averaging motifs into two classes based on the type of fluctuations in the motif counts.

For a given motif *H*, let ${H}_{1},\ldots ,{H}_{m}$ denote all possible motifs that can be constructed by merging two copies of *H* at one or more vertices. We can then write the variance of the motif count as (see^37,40–42^ and the Methods section)

8 $${\rm{Var}}\,(N(H))={C}_{1}{\mathbb{E}}\,[N({H}_{1})]+\cdots +{C}_{m}{\mathbb{E}}\,[N({H}_{m})]+{\mathbb{E}}\,{[N(H)]}^{2}O({n}^{-1}).$$

for constants ${C}_{1},\ldots ,{C}_{m}$. Using (8), we can determine for any motif *H* whether it is self-averaging or not. First, we find all motifs that are created by merging two copies of *H*. For the triangle motif for example, these motifs are the bow-tie, where two triangles are merged at one single vertex, the diamond of Fig. 3b, and the triangle itself. We find the order of magnitude of the expected number of these motifs using Theorem 1 to obtain the variance of *N*(*H*). We divide by ${\mathbb{E}}\,{[N(H)]}^{2}$, also obtained by Theorem 1, and check whether this fraction is diverging or not. Table 1 shows for which values of $\tau \in (2,3)$ the motifs on 3, 4 and 5 vertices are self-averaging. For example, the triangle turns out to be self-averaging only for $\tau \in (2,5/2)$.

**Table 1:** The values of *τ* ∈ (2, 3) where the motifs of Figs 1 and 2 are self-averaging.

Self-averaging for	Subfigs of Fig. 1	Subfigs of Fig. 2
(2, 3)	c	a, b, c, d
(2, 5/2)	a	f, g, l, o
(2, 7/3)		i
—	b, d, e, f, g, h	e, h, j, k, m, n, p, q, r, s, t, u

Here is a general observation that underlines the importance of the dominant motif structure:

#### Theorem 2.

*All self-averaging motifs for any* $\tau \in (2,3)$ *have dominant free variation structures that consist of vertices with hidden variables* ${\rm{\Theta }}(\sqrt{n})$ *only*.

We prove this theorem in Supplementary Material 5. Note that the condition on the dominant motif structure is a necessary condition for being self-averaging, but it is not a sufficient one, as the triangle example shows. Table 1 shows the values of $\tau$ for which all connected motifs on 3, 4 and 5 vertices are self-averaging. Combining the classification of the motifs into self-averaging and non-self-averaging with the classification based on the value of *B*_*f*_(*H*) from (6) as well as the difference between the expected and typical number of motifs yields a classification into the following three types of motifs:

#### Type I: Motifs with small variance. ${B}_{f}(H)=0\,{\rm{and}}\,{\rm{Var}}\,(N(H))/{\mathbb{E}}\,{[N(H)]}^{2}\to 0$

**Figure 4:**
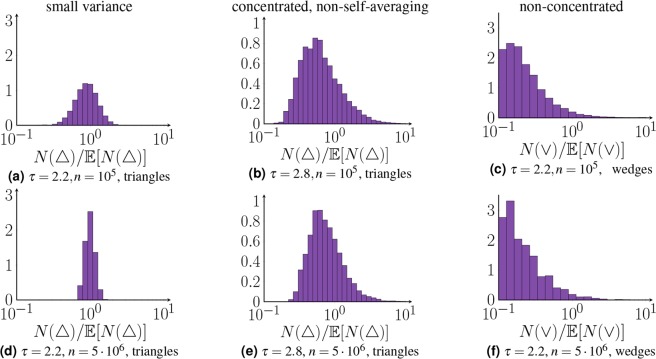
Density approximation of the normalized triangle and wedge counts for various values of $\tau$ and *n*, obtained over 10^4^ network samples.

These motifs only contain vertices of degrees ${\rm{\Theta }}(\sqrt{n})$. The number of such rescaled motifs converges to a constant^43^. Furthermore, the variance of the number of motifs is small compared to the second moment, so that the fluctuations of these types of motifs are quite small and vanish in the large network limit. The triangle for $\tau  < 5/2$ is an example of such a motif, shown in Fig. 4b,e.

#### Type II: Concentrated, non-self-averaging motifs. ${B}_{f}(H)=0\,{\rm{and}}\,{\rm{Var}}\,(N(H))/{\mathbb{E}}\,{[N(H)]}^{2} \nrightarrow 0$

These motifs also only contain vertices of degrees $\sqrt{n}$. Again, the rescaled number of such motifs converges to a constant in probability^43^. Thus, most network samples contain a similar amount of motifs as *n* grows large, even though these motifs are non-self-averaging. Still, in rare network samples the number of motifs significantly deviates from its typical number, causing the variance of the number of motifs to be large. Figure 4a,d illustrate this for triangle counts for $\tau \ge 5/2$. The fluctuations are larger than for the concentrated motifs, but most of the samples have motif counts close to the expected value.

#### Type III: Non-concentrated motifs. *B*_*f*_ (*H*) > 0

These motifs contain hub vertices. The expected and typical number of such motifs therefore scale differently in *n*. By Theorem 2, these motifs are non-self-averaging. The rescaled number of such motifs may not converge to a constant, so that two network samples contain significantly different motif counts. Figure 4c,f show that the fluctuations of these motifs are indeed of a different nature, since most network samples have motif counts that are far from the expected value.

### Data

**Figure 5:**
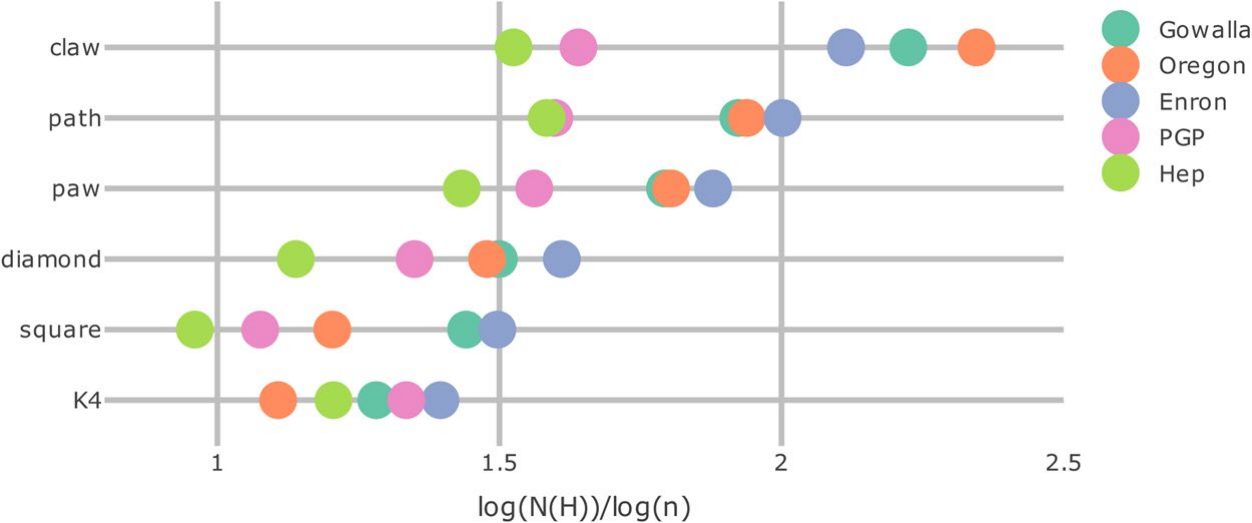
Number of graphlets on four vertices in five data sets on logarithmic scale: $\mathrm{log}(N(H))$/$\mathrm{log}(n)$. The ordering of the six different graphlets is from the most occurring in the hidden-variable model to the least. The values of the graphlet counts are presented in Supplementary Table 1.

We now investigate motifs in five real-world networks with heavy-tailed degree distributions: the Gowalla social network^44^, the Oregon autonomous systems network^44^, the Enron email network^44,45^, the PGP web of trust^46^ and the High Energy Physics collaboration network (HEP)^44^. Table 2 provides detailed statistics of these data sets. Because the number of motifs can be obtained from the number of graphlets, we focus on graphlet counts. Figure 5 shows the graphlet counts on a logarithmic scale. The order of the graphlets is from the most occurring graphlet (the claw), to the least occurring graphlet (the square and *K*_4_) in the hidden-variable model, see Supplementary Fig. 2. In three networks the motif ordering follows that of the hidden-variable model, while in two networks the ordering is different. In the HEP collaboration network, for example, *K*_4_ occurs more frequently than the square. While this is not predicted by the hidden-variable model, it naturally arises due to the frequently occurring collaboration between four authors, which creates *K*_4_ instead of the square. It would be interesting to see if this deviation from the ordering of the hidden-variable model can be linked to the specific nature of the data set in other examples.

**Table 2:** Statistics of the five data sets, where *n* is the number of vertices, *m* the number of edges, and *τ* the power-law exponent fitted by the procedure of ^5^.

	*n*	*m*	*τ*
Gowalla	196591	950327	2.65
Oregon	11174	23409	2.08
Enron	36692	183831	1.97
PGP	10680	24316	2.24
Hep	9877	25998	3.50

**Figure 6:**
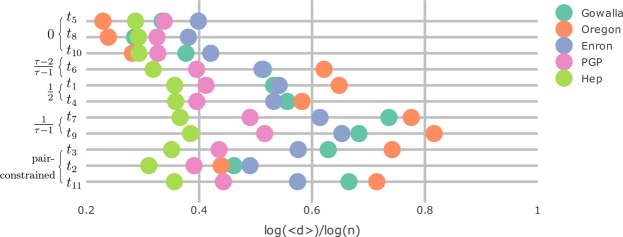
Average degree of the vertex types displayed in Supplementary Fig. 2 in 5 data sets on logarithmic scale. The curly brackets indicate the typical degree exponent of the vertex type in the hidden-variable model.

Supplementary Fig. 2 enumerates all possible vertex types in graphlets on 4 vertices. In the hidden-variable model, vertex types *t*_7_ and *t*_9_ have typical degrees proportional to ${n}^{1/(\tau -1)}$, vertex types *t*_1_ and *t*_4_ typically have degrees proportional to $\sqrt{n}$, vertex type *t*_6_ typically has degree proportional to ${n}^{(\tau -2)/(\tau -1)}$ and vertex types *t*_5_, *t*_8_, *t*_10_, *t*_11_ typically have constant degree. vertex types *t*_2_, *t*_3_ and *t*_11_ do not have a unique optimizer. The degrees of these vertex types are pair-constrained (see the proof of Lemma 3). Figure 6 shows the typical degree of all 11 vertex types in the five real-world data sets. Vertices with typical degree 1 in the hidden-variable model have the lowest degree in the five data sets. Vertices that have typical degree ${n}^{1/(\tau -1)}$ in the hidden-variable model also have the highest degree among all vertex types in these five real-world data sets. Thus, typical degrees of vertices in a graphlet roughly follow the same ordering as in the hidden-variable model in these data sets.

The High Energy Physics collaboration network does not have a large distinction between the degrees of the different vertex types. This may be related to the fact that this network has less heavy-tailed degrees than the other networks (see Table 2).

## Discussion

By developing a variational principle for the dominant degree composition of motifs in the hidden-variable model, we have identified the asymptotic growth of motif counts and their fluctuations for all motifs. This allowed us to determine for which values of the degree exponent $\tau \in (2,3)$ the number of motifs is self-averaging. We further divide the non-self-averaging motifs into two classes with substantially different concentration properties.

Hub vertices in dominant motif structures cause wild degree fluctuations and non-self-averaging behavior, so that large differences between the average motif count and the motif count in one sample of the random network model arise. Non-self-averaging motifs without a hub vertex show milder fluctuations.

We expect that the variational principle can be extended to different random graph models, such as the hyperbolic random graph, the preferential attachment model and random intersection graphs. For example, for the hyperbolic random graph, the dominant structure of complete graphs is known to be $\sqrt{n}$ degrees^47^ like in the hidden-variable model, but the dominant structures of other motifs are yet unknown.

In this paper, we presented a case study for motifs on 4 vertices in five scale-free network data sets. It would be interesting to perform larger network data experiments to investigate whether motifs in real-world network data also have typical vertex degrees, and to what extent these vertex degrees are similar to the ones of the hidden-variable model. Similarly, investigating the typical behavior of motifs on more than 4 vertices in real-world data compared to the hidden-variable model is another topic for future research.

It would also be interesting to develop statistical tests for the presence of motifs in real-world data using the results from this paper. For example, one could compare the ordering of all motifs for size *k* from the most frequent occurring to the least frequent occurring motif, and compare this to the ordering in a hidden-variable with the same degree-exponent. This could shed some light on which motifs in a given data set appear more often than expected.

## Methods

### Fluctuations

#### Triangle fluctuations

We first illustrate how we can apply the variational principle to obtain the variance of the number of subgraphs by computing the variance of the number of triangles in the hidden-variable model. Let Δ denote the number of triangles, and let ${{\rm{\Delta }}}_{i,j,k}$ denote the event that vertices *i*, *j* and *k* form a triangle. Then, we can write the number of triangles as

9 $${\rm{\Delta }}=\frac{1}{6}\,\mathop{{\sum }^{^{\prime} }}\limits_{i,j,k\in [n]}\,{{\mathbb{1}}}_{{{\rm{\Delta }}}_{i,j,k}},$$

where $\sum ^{\prime}$ denotes the sum over distinct indices. Thus, the variance of the number of triangles can be written as

10 $${\rm{Var}}\,({\rm{\Delta }})=\mathop{{\sum }^{^{\prime} }}\limits_{i,j,k\in [n]}\,\mathop{{\sum }^{^{\prime} }}\limits_{s,t,u\in [n]}\,{\mathbb{P}}\,({{\rm{\Delta }}}_{i,j,k},{{\rm{\Delta }}}_{s,t,u})-{\mathbb{P}}\,({{\rm{\Delta }}}_{i,j,k})\,{\mathbb{P}}\,({{\rm{\Delta }}}_{s,t,u}).$$

When *i*, *j*, *k* and *s*, *t*, *u* do not overlap, the hidden variables of *i*, *j*, *k* and *s*, *t*, *u* are independent, so that the event that *i*, *j* and *k* form a triangle and the event that *s*, *t* and *u* form a triangle are independent. Thus, when *i*, *j*, *k*, *s*, *t*, *u* are all distinct, ${\mathbb{P}}\,({{\rm{\Delta }}}_{i,j,k},{{\rm{\Delta }}}_{s,t,u})={\mathbb{P}}\,({{\rm{\Delta }}}_{i,j,k})\,{\mathbb{P}}\,({{\rm{\Delta }}}_{s,t,u})$, so that the contribution from 6 distinct indices to (10) is zero. On the other hand, when $i=u$ for example, the first term in (10) denotes the probability that a bow tie (see Fig. 2i) is present with *i* as middle vertex. Furthermore, since the degrees are i.i.d. and the edge statuses are independent as well, ${\mathbb{P}}\,({{\rm{\Delta }}}_{i,j,k})$ is the same for any $i\ne j\ne k$, so that

11 $${\mathbb{P}}\,({{\rm{\Delta }}}_{i,j,k})=\frac{{\mathbb{E}}\,[{\rm{\Delta }}]}{6(\begin{array}{c}n\\ 3\end{array})}=\frac{{\mathbb{E}}\,[{\rm{\Delta }}]}{6{n}^{3}}(1+o(1)).$$

This results in

12 $$\begin{array}{rcl}{\rm{Var}}\,({\rm{\Delta }}) & = & 9{\mathbb{E}}\,[\#\,\mathrm{bow} \mbox{-} \mathrm{ties}]-9{n}^{-1}{\mathbb{E}}\,{[{\rm{\Delta }}]}^{2}+18{\mathbb{E}}\,[\#\,{\rm{diamonds}}]\\  &  & -\,18{n}^{-2}{\mathbb{E}}\,{[{\rm{\Delta }}]}^{2}+6{\mathbb{E}}\,[{\rm{\Delta }}]-6{n}^{-3}{\mathbb{E}}\,{[{\rm{\Delta }}]}^{2}\\  & = & 9{\mathbb{E}}\,[\#\,\mathrm{bow} \mbox{-} \mathrm{ties}]+18{\mathbb{E}}\,[\#\,{\rm{diamonds}}]\\  &  & +\,6{\mathbb{E}}\,[{\rm{\Delta }}]+{\mathbb{E}}\,{[{\rm{\Delta }}]}^{2}O({n}^{-1}),\end{array}$$

where the diamond motif is as in Fig. 3b. The combinatorial factors 9, 18 and 6 arise because there are 9 ways to construct a bow tie (18 for a diamond, and 6 for a triangle) by letting two triangles overlap. The diamond motif does not satisfy the assumption in Theorem 1 that the optimal solution to (6) is unique. However, we can show the following result:

##### **Lemma 3.**

$${\mathbb{E}}\,[{\rm{number}}\,{\rm{of}}\,{\rm{diamonds}}]={\rm{\Theta }}\,({n}^{6-2\tau })\,\mathrm{log}(n)$$.

*Proof*. Let *i* and *j* be the vertices at the diagonal of the diamond, and *k* and *s* the corner vertices. Then (5) is optimized for ${\alpha }_{i}=\beta$, ${\alpha }_{j}=1-\beta$, ${\alpha }_{k}=\beta$ and ${\alpha }_{s}=1-\beta$ for all values of $\beta \in [1/2,1]$ (see Supplementary Information 6). All these optimizers together give the major contribution to the number of diamonds. Thus, we need to find the number of sets of four vertices, satisfying

13 $${h}_{i}{h}_{j}={\rm{\Theta }}(n),\,{h}_{i} > {h}_{j},\,{h}_{k}={\rm{\Theta }}({h}_{i}),\,{h}_{s}={\rm{\Theta }}({h}_{j}).$$

Given *h*_*i*_ and *h*_*j*_, the number of sets of two vertices *k*, *s* with ${h}_{k}={\rm{\Theta }}({h}_{i})$ and ${h}_{s}={\rm{\Theta }}({h}_{j})$ is given by ${n}^{2}{h}_{i}^{1-\tau }{h}_{j}^{1-\tau }={\rm{\Theta }}({n}^{3-\tau })$, where we used that ${h}_{i}{h}_{j}={\rm{\Theta }}(n)$. The number of sets of vertices *i*, *j* such that ${h}_{i}{h}_{j}={\rm{\Theta }}(n)$ can be found using that the product of two independent power-law random variables is again distributed as a power law, with an additional logarithmic term^48^, Eq. (2.16) (where in our setting equality holds in^48^, Eq. (2.16), since we assume a pure power-law distribution). Thus, the number of sets of vertices with ${h}_{i}{h}_{j}={\rm{\Theta }}(n)$ scales as ${n}^{2}{n}^{1-\tau }\,\mathrm{log}(n)$. Then, the expected number of sets of four vertices satisfying all constraints on the degrees scales as ${n}^{6-2\tau }\,\mathrm{log}(n)$. By (4), the probability that a diamond exists on degrees satisfying (13) is asymptotically constant, so that the expected number of diamonds also scales as ${n}^{6-2\tau }\,\mathrm{log}(n)$.$\square$

Theorem 1 gives for the number of bow ties that (using^49^ to find the optimal partition)

14 $${\mathbb{E}}\,[\#\,{\rm{bow}}\,{\rm{ties}}]=\{\begin{array}{ll}{\rm{\Theta }}({n}^{\tfrac{5}{2}(3-\tau )}) & \tau  < \tfrac{7}{3},\\ {\rm{\Theta }}({n}^{4-\tau }) & \tau \ge \tfrac{7}{3},\end{array}$$

and for the number of triangles (again using^49^) that ${\mathbb{E}}\,[{\rm{\Delta }}]={\rm{\Theta }}({n}^{3(3-\tau )/2})$. Combining this with (12) results in

15 $${\rm{Var}}\,({\rm{\Delta }})=\{\begin{array}{ll}{\rm{\Theta }}({n}^{\tfrac{5}{2}(3-\tau )}) & \tau  < \tfrac{7}{3},\\ {\rm{\Theta }}({n}^{4-\tau }) & \tau \ge \tfrac{7}{3}.\end{array}$$

To investigate whether the triangle motif is self-averaging, we need to compare the variance to the second moment of the number of triangles, which results in

16 $$\frac{{\rm{Var}}\,({\rm{\Delta }})}{{\mathbb{E}}\,{[{\rm{\Delta }}]}^{2}}=\{\begin{array}{ll}{\rm{\Theta }}({n}^{\tfrac{1}{2}(\tau -3)}), & \tau  < \tfrac{7}{3},\\ {\rm{\Theta }}({n}^{2\tau -5}), & \tau \ge \tfrac{7}{3}.\end{array}$$

Therefore,

17 $$\mathop{\mathrm{lim}}\limits_{n\to \infty }\,\frac{{\rm{Var}}\,({\rm{\Delta }})}{{\mathbb{E}}\,{[{\rm{\Delta }}]}^{2}}=\{\begin{array}{ll}0 & \tau  < \tfrac{5}{2},\\ \infty  & \tau  > \tfrac{5}{2}.\end{array}$$

For $\tau =5/2$, the limit in (17) is of constant order of magnitude. Thus, the number of triangles is self-averaging as long as $\tau  < \tfrac{5}{2}$. When $\tau \ge \tfrac{5}{2}$ the number of triangles is not self-averaging.

#### General motif fluctuations

We now compute the variance of general motifs, similar to the triangle example. Let ${\boldsymbol{i}}=({i}_{1},\ldots ,{i}_{k})$ be such that ${i}_{p}\ne {i}_{q}$ when $p\ne q$. We can then write the variance as

18 $$\begin{array}{rcl}{\rm{Var}}\,(N(H)) & = & \sum _{{\boldsymbol{i}}\in {[n]}^{k}}\,\sum _{{\boldsymbol{j}}\in {[n]}^{k}}\,({\mathbb{P}}\,({H}_{{\boldsymbol{i}}},{H}_{{\boldsymbol{j}}}\,{\rm{present}})\\  &  & -\,{\mathbb{P}}\,({H}_{{\boldsymbol{i}}}\,{\rm{present}})\,{\mathbb{P}}\,({H}_{{\boldsymbol{j}}}\,{\rm{present}})).\end{array}$$

The sum splits into several cases, depending on the overlap of ***i*** and ***j***. The term where ***i*** and ***j*** do not overlap equals zero, since edges between vertices that do not overlap are independent.

Now suppose ***i*** and ***j*** overlap at $${i}_{{t}_{1}},\ldots {i}_{{t}_{r}}$$ and $${j}_{{s}_{1}},\ldots ,{j}_{{s}_{r}}$$ for some $$r > 0$$. Then $${\mathbb{P}}\,({H}_{{\boldsymbol{i}}},{H}_{{\boldsymbol{j}}}\,{\rm{present}})$$ is equal to the probability that motif $$\tilde{H}$$ is present on vertices $${i}_{1},\ldots ,{i}_{k}$$, $${j}_{1},\ldots ,{j}_{k}$$\$${j}_{{s}_{1}},\ldots ,{j}_{{s}_{r}}$$, where $$\tilde{H}$$ denotes the motif that is constructed by merging two copies of *H* at $${i}_{{t}_{1}}$$ with $${j}_{{s}_{1}}$$, at $${i}_{{t}_{2}}$$ with $${j}_{{s}_{2}}$$ and so on. Thus, this term can be written as

19 $$\mathop{{\sum }^{^{\prime} }}\limits_{{t}_{1},\ldots ,{t}_{2k-r}}\,{\mathbb{P}}\,({\tilde{H}}_{{t}_{1},\ldots ,{t}_{2k-r}}\,{\rm{present}})={\mathbb{E}}\,[N(\tilde{H})],$$

where $\sum ^{\prime}$ denotes a sum over distinct indices. Furthermore, since the degrees are i.i.d. as well as the connection probabilities, $${\mathbb{P}}\,({H}_{{\boldsymbol{i}}}\,{\rm{present}})={\mathbb{E}}\,[N(H)]/(\begin{array}{c}n\\ k\end{array})$$. Thus,

20 $$\mathop{{\sum }^{^{\prime} }}\limits_{{t}_{1},\ldots ,{t}_{2k-r}}\,{\mathbb{P}}\,({H}_{{t}_{1},\ldots ,{t}_{k}}\,{\rm{present}})\,{\mathbb{P}}\,({H}_{{t}_{k-r},\ldots ,{t}_{2k-r}}\,{\rm{present}})={n}^{-r}{\mathbb{E}}\,{[N(H)]}^{2}O\mathrm{(1).}$$

Let ${H}_{1},\ldots ,{H}_{l}$ denote all motifs that can be constructed by merging two copies of *H* at at least one vertex. We can then write the variance of the motif count as (see^37,40–42^)

21 $${\rm{Var}}\,(N(H))={C}_{1}{\mathbb{E}}\,[N({H}_{1})]+\cdots +{C}_{l}{\mathbb{E}}\,[N({H}_{l})]+{\mathbb{E}}\,{[N(H)]}^{2}O({n}^{-1}).$$

where *C*_*i*_ is a combinatorial constant that denotes the number of distinct ways to merge two copies of *H* into *H*_*i*_. These constants satisfy^40^

22 $$\sum _{i=1}^{l}\,{C}_{i}=\sum _{s=0}^{k-1}\,{(\begin{array}{c}k\\ s\end{array})}^{2}(k-s)!.$$

## Supplementary information

Supplementary information
