# Supplementary material for: Variational principle for scale-free network motifs
Source: Sci Rep. 2019 May 1;9:6762. doi: 10.1038/s41598-019-43050-8 (PMC6494877; doi:10.1038/s41598-019-43050-8)
Supplement: Supplementary file 1 — Supplementary information [file 41598_2019_43050_MOESM1_ESM.pdf]

Supplementary Material to ‘Variational principle for scale-free network motifs’

Clara Stegehuis, Remco van der Hofstad, Johan S.H. van Leeuwaarden

1 Additional motif structures

Figure 1 presents the typical dominant motif structures for all connected motifs on 5 vertices.

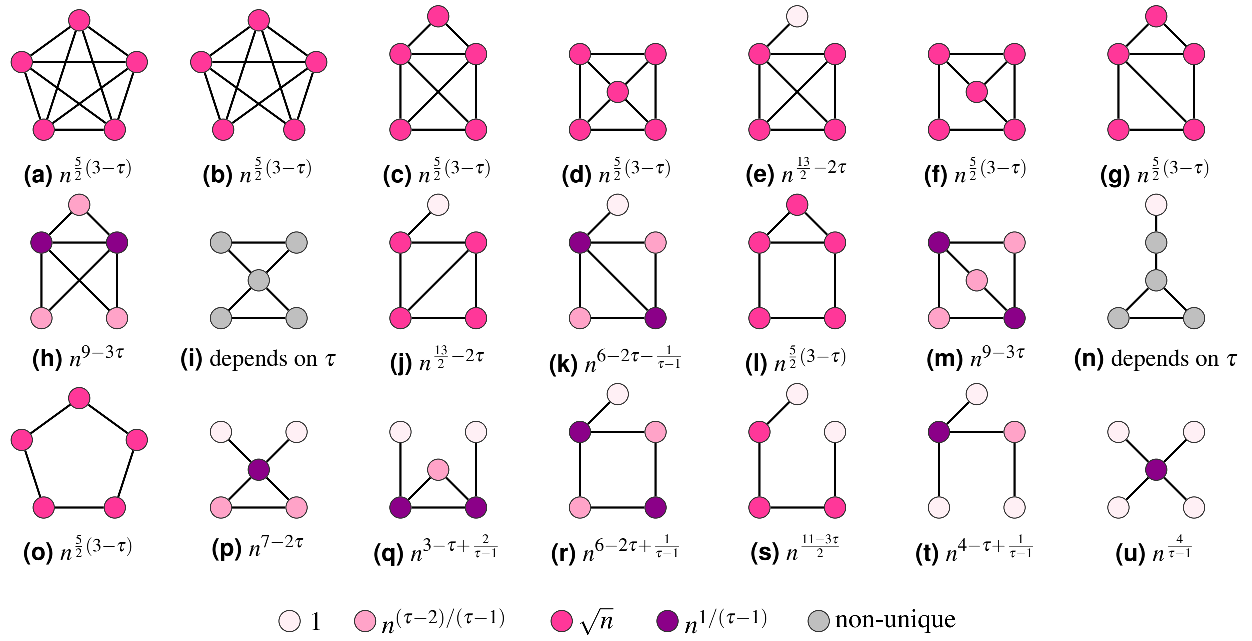

Supplementary Figure 1. Typical magnitude of the number of motifs on 5 vertices. The vertex color indicates the dominant vertex degree.

2 Proof of Theorem 1

We now investigate the relation between the expected number of motifs and the optimization problem

$$B_f(H) = \max_{\mathscr{P}} \left[|S_1| - |S_2| - \frac{2E_{S_1} + E_{S_1,S_3}}{\tau - 1} \right]. \tag{1}$$

Let $N(H, \boldsymbol{\alpha}, \varepsilon)$ denote the number of times motif H occurs on vertices with degrees $[\varepsilon, 1/\varepsilon](n^{\alpha_i})_{i\in[k]}$ and let $\boldsymbol{\alpha}^*$ be defined as

$$\alpha_i^* = \begin{cases} 0 & \text{if } i \in S_1, \\ 1 & \text{if } i \in S_2, \\ \frac{1}{2} & \text{if } i \in S_3. \end{cases} \tag{2}$$

Then, the following theorem provides a more detailed version of Theorem 1:

Theorem 1 (General motifs, expectation)**.** *Let H be a motif on k vertices such that the solution to (1) is unique. Then, for any $\boldsymbol{\alpha} \neq \boldsymbol{\alpha}^*$ and $0 < \varepsilon < 1$,*

$$\frac{\mathbb{E}\left[N(H,\boldsymbol{\alpha},\varepsilon)\right]}{\mathbb{E}\left[N(H,\boldsymbol{\alpha}^*,\varepsilon)\right]} \to 0. \tag{3}$$

Furthermore,

$$\frac{\mathbb{E}\left[N(H,\boldsymbol{\alpha}^*,\varepsilon)\right]}{n^{\frac{3-\tau}{2}k+\frac{\tau-2}{2}B(H)}} = f(\varepsilon)\Theta(1) \tag{4}$$

for some function $f(\varepsilon)$ not depending on n.

We now prove Theorem 1. In the hidden-variable model, the connection probability of vertices with hidden variables h_i and h_j equals

$$p(h_i,h_j) = \min(h_ih_j/(\mu n),1). \tag{5}$$

Assume that $h_i = \Theta(n^{\alpha_i})$ for some $\alpha_i \geq 0$ for all i. Then, the probability that motif H exists on vertices $(v_i)_{i\in[k]}$ with hidden variables $\boldsymbol{h} = (n^{\alpha_i})_{i\in[k]}$ satisfies

$$\mathbb{P}\left(H \text{ present on } (v_1,\cdots,v_k) \mid \boldsymbol{h} = (n^{\alpha_1})_{i\in[k]}\right) = \Theta\left(\prod_{(v_i,v_j)\in E_H:\alpha_i+\alpha_j<1} n^{\alpha_i+\alpha_j-1}\right). \tag{6}$$

The hidden variables are an i.i.d. sample from a power-law distribution. Therefore,

$$\mathbb{P}\left(h_i \in [\varepsilon,1/\varepsilon](\mu n)^{\alpha}\right) = \int_{\varepsilon(\mu n)^{\alpha}}^{1/\varepsilon(\mu n)^{\alpha}} cx^{-\tau}\mathrm{d}x = K(\varepsilon)(\mu n)^{\alpha(1-\tau)} \tag{7}$$

for some constant $K(\varepsilon)$ not depending on n. The expected number of vertices with degrees in $[\varepsilon,1/\varepsilon](\mu n)^{\alpha}$ scales as $\Theta(n^{(1-\tau)\alpha+1})$. Then, the number of sets of vertices with degrees in $[\varepsilon,1/\varepsilon](n^{\alpha_i})_{i\in[k]}$ scales as

$$\Theta\left(n^{k+(1-\tau)\sum_i\alpha_i}\right). \tag{8}$$

Combining (6) and (8) yields that the contribution from vertices with degrees $n^{\boldsymbol{\alpha}} = (n^{\alpha_i})_{i\in[k]}$ to the expected number of motifs, $\mathbb{E}\left[N(H,\boldsymbol{\alpha}),\varepsilon\right]$ is

$$\mathbb{E}\left[N(H,\boldsymbol{\alpha},\varepsilon)\right] = \Theta\left(n^{k+(1-\tau)\sum_i\alpha_i}\prod_{(i,j)\in E_H:\alpha_i+\alpha_j<1} n^{\alpha_1+\alpha_j-1}\right). \tag{9}$$

The maximum contribution is obtained for α_i that maximize

$$\max(1-\tau)\sum_i\alpha_i + \sum_{(i,j)\in E_H:\alpha_i+\alpha_j<1} \alpha_i+\alpha_j-1 \tag{10}$$

for $\alpha_i \geq 0$. The following lemma shows that this optimization problem attains its maximum for highly specific values of α:

Lemma 2 (Maximum contribution to expected number of motifs)**.** *Let H be a connected graph on k vertices. If the solution to (10) is unique, then the optimal solution satisfies $\alpha_i \in \{0,\frac{1}{2},1\}$ for all i. If it is not unique, then there exist at least 2 optimal solutions with $\alpha_i \in \{0,\frac{1}{2},1\}$ for all i.*

Proof. Defining $\beta_i = \alpha_i - \frac{1}{2}$ yields for (10)

$$\max\frac{1-\tau}{2}k + (1-\tau)\sum_i\beta_i + \sum_{(i,j)\in E_H:\beta_i+\beta_j<0} \beta_i+\beta_j, \tag{11}$$

over all possible values of $\beta_i \geq -\frac{1}{2}$. Then, we have to prove that $\beta_i \in \{-\frac{1}{2},0,\frac{1}{2}\}$ for all i in the optimal solution. Note that (11) is a piecewise linear function in β. Therefore, if (11) has a unique maximum, it must be attained at the boundary for β_i or at a

border of one of the linear sections. Thus, any unique optimal value of β_i satisfies $\beta_i = -\frac{1}{2}$ or $\beta_i + \beta_j = 0$ for some j. Note that this implies that $\beta_i \leq \frac{1}{2}$. We ignore the constant factor of $(1-\tau)\frac{k}{2}$ in (11), since it does not influence the optimal β values. Rewriting (11) without the constant factor yields

$$\max \sum_i \beta_i \left(1 - \tau + \#\{\text{ edges to } j \text{ with } \beta_j < -\beta_i\}\right). \tag{12}$$

The proof of the lemma then consists of two steps:
Step 1. Show that any unique solution does not have vertices i with $|\beta_i| \in (0, \frac{1}{2})$.
Step 2. Show that any optimal solution that is not unique can be transformed into two different optimal solutions with $\beta_i \in \{-\frac{1}{2}, 0, \frac{1}{2}\}$ for all i.

Step 1. We show that when the solution to (12) is unique, it is never optimal to have $|\beta| \in (0, \frac{1}{2})$. Let

$$\tilde{\beta} = \min_{i:|\beta_i|>0} |\beta_i|. \tag{13}$$

If $\tilde{\beta} = \frac{1}{2}$, then we are finished, so assume that $\tilde{\beta} < \frac{1}{2}$. Then, there exist $N_{\tilde{\beta}^-}$ vertices with their β value equal to $-\tilde{\beta}$, and $N_{\tilde{\beta}^+}$ vertices with value $\tilde{\beta}$, where $N_{\tilde{\beta}^+} + N_{\tilde{\beta}^-} \geq 1$. Furthermore, let $E_{\tilde{\beta}^-}$ denote the number of edges from vertices with value $-\tilde{\beta}$ to other vertices j such that $\beta_j < \tilde{\beta}$, and $E_{\tilde{\beta}^+}$ the number of edges from vertices with value $\tilde{\beta}$ to other vertices j such that $\beta_j < -\tilde{\beta}$. Then, the contribution from these vertices to (12) is

$$\tilde{\beta}\left((1-\tau)\left(N_{\tilde{\beta}^+} - N_{\tilde{\beta}^-}\right) + E_{\tilde{\beta}^+} - E_{\tilde{\beta}^-}\right). \tag{14}$$

Because the optimizer of (12) is unique, the term inside the brackets cannot equal zero. Then, increasing $\tilde{\beta}$ if the term inside brackets is larger than zero, or decreasing it if it is smaller than zero would improve the optimal contribution, which is not possible. Thus, if the optimal solution is unique, we must have $\tilde{\beta} = \frac{1}{2}$. This shows that any unique solution satisfies $\beta_i \in \{-\frac{1}{2}, 0, \frac{1}{2}\}$ for all i.

Step 2. If the solution to (12) is not unique, then by the same argument that leads to (14), there exist $\hat{\beta}_1, \ldots, \hat{\beta}_s > 0$ for some $s \geq 1$ such that

$$\hat{\beta}_j\left((1-\tau)\left(N_{\hat{\beta}_j^+} - N_{\hat{\beta}_j^-}\right) + E_{\hat{\beta}_j^+} - E_{\hat{\beta}_j^-}\right) = 0 \quad \forall j \in [s]. \tag{15}$$

Here we use the same notation as in (14). All other values of β must either be $0, \frac{1}{2}$ or $-\frac{1}{2}$ by the argument in Step 3. Thus, setting all $\hat{\beta}_j$ to zero does not change the value of the solution, and setting all $\hat{\beta}_j$ to $\frac{1}{2}$ also does not change the value of the solution. Thus, if the solution to (12) is not unique, at least 2 solutions exist with $\beta_i \in \{-\frac{1}{2}, 0, \frac{1}{2}\}$ for all i. □

Completion of the proof of Theorem 1. We first rewrite (10) using Lemma 2. By Lemma 2, the maximal value of (10) is attained by partitioning V_H into the sets S_1, S_2, S_3 such that vertices in S_1 have $\alpha_i = 0$, vertices in S_2 have $\alpha_i = 1$ and vertices in S_3 have $\alpha_i = \frac{1}{2}$. Then, the edges with $\alpha_i + \alpha_j < 1$ are edges inside S_1 and edges between S_1 and S_3. If we denote the number of edges inside S_1 by E_{S_1} and the number of edges between S_1 and S_3 by E_{S_1,S_3}, then we can rewrite (10) as

$$\max_{\mathscr{P}} \,(1-\tau)\left(|S_2| + \tfrac{1}{2}|S_3|\right) - E_{S_1} - \tfrac{1}{2}E_{S_1,S_3} \tag{16}$$

over all partitions $\mathscr{P}$ of the vertices of H into S_1, S_2, S_3. Using that $|S_3| = k - |S_1| - |S_2|$ yields

$$\max_{\mathscr{P}} \frac{1-\tau}{2}k + \frac{\tau-1}{2}\left(|S_1| - |S_2| - \frac{2E_{S_1} + E_{S_1,S_3}}{\tau-1}\right), \tag{17}$$

Since k is fixed and $\tau - 1 > 0$, maximizing (10) is equivalent to maximizing

$$B_f(H) = \max_{\mathscr{P}} \left[|S_1| - |S_2| - \frac{2E_{S_1} + E_{S_1,S_3}}{\tau - 1}\right]. \tag{18}$$

Furthermore, by Lemma 2, the optimal value of (18) is unique if and only if the solution to (10) is unique.

Let $\boldsymbol{\alpha}^*$ be the unique optimizer of (10). Then, by (9), for any $\boldsymbol{\alpha} \neq \boldsymbol{\alpha}^*$

$$\frac{\mathbb{E}\left[N(H,\boldsymbol{\alpha},\varepsilon)\right]}{\mathbb{E}\left[N(H,\boldsymbol{\alpha}^*,\varepsilon)\right]} = \Theta\left(n^{-\eta}\right) \tag{19}$$

for some $\eta > 0$. Combining this with (18) proves the first part of the theorem. By (9), the contribution of the maximum is then given by

$$\mathbb{E}\left[N(H,\boldsymbol{\alpha}^*,\varepsilon)\right] = n^k n^{\frac{1-\tau}{2}(k+B_f(H))} = n^{\frac{3-\tau}{2}k+\frac{\tau-1}{2}B_f(H)}, \tag{20}$$

which proves the second part of the theorem.

3 Typical motif counts

We now present equivalent versions of Lemma 2 and Theorem 1 for typical motif counts:

Lemma 3 (Maximum contribution to typical number of motifs)**.** *Let H be a connected graph on k vertices. If the solution to (10) with the extra constraint $\alpha_i \in [0, 1/(\tau-1)]$ for all i is unique, then the optimal solution satisfies $\alpha_i \in \{0, (\tau-2)/(\tau-1), \frac{1}{2}, 1/(\tau-1)\}$ for all i. Furthermore, $\alpha_i = 0$ if and only if the degree of vertex i in H equals 1. If the solution is not unique, then there exist at least 2 optimal solutions with $\alpha_i \in \{0, (\tau-2)/(\tau-1), \frac{1}{2}, 1/(\tau-1)\}$ for all i.*

Thus, the optimization problem can be translated into the optimal partition $\mathscr{P}$ of all vertices of H with degree at least 2 into three sets, S_1, S_2 and S_3. The vertices in S_1 correspond to vertices that have degree proportional to $n^{(\tau-2)/(\tau-1)}$ in the graph, vertices in S_2 correspond to the maximal degree vertices with degrees proportional to $n^{1/(\tau-1)}$, and S_3 corresponds to the vertices of $\sqrt{n}$ degrees.

Let $E_{S_i,1}$ denote the number of edges between vertices in S_i and vertices with degree 1 in H. Then, rewriting the optimal solution of (10) results in

$$\max_{\mathscr{P}} (1-\tau)\left(\frac{\tau-2}{\tau-1}|S_1| + \frac{1}{\tau-1}|S_2| + \tfrac{1}{2}|S_3|\right)\frac{\tau-3}{\tau-1}E_{S_1} + \frac{\tau-3}{2(\tau-1)}E_{S_1,S_3} - \frac{E_{S_1,1}}{\tau-1} - \frac{\tau-2}{\tau-1}E_{S_2,1} - \frac{1}{2}E_{S_3,1}, \tag{21}$$

over all partitions $\mathscr{P}$ of the vertices of H with degree at least 2 into S_1, S_2, S_3. Using that $|S_3| = k - |S_1| - |S_2| - k_1$, $E_{S_3,1} = k_1 - E_{S_1,1} - E_{S_2,1}$, where k_1 denotes the number of degree 1 vertices in H, and extracting a factor $(3-\tau)/2$ results in

$$\max_{\mathscr{P}} \frac{1-\tau}{2}k + \frac{(3-\tau)}{2}\left(|S_1| - |S_2| + \frac{\tau-2}{3-\tau}k_1 - \frac{2E_{S_1}+E_{S_1,S_3}}{\tau-1} - \frac{E_{S_1,1}-E_{S_2,1}}{\tau-1}\right), \tag{22}$$

Since k and k_1 are fixed and $3-\tau > 0$, we need to maximize

$$B_t(H) = \max_{\mathscr{P}} |S_1| - |S_2| - \frac{2E_{S_1}+E_{S_1,S_3}+E_{S_1,1}-E_{S_2,1}}{\tau-1}. \tag{23}$$

Theorem 4 (General motifs, typical)**.** *Let H be a motif on k vertices such that the solution to (23) is unique and let k_1 denote the number of vertices of degree 1 in H. With high probability, the number of motifs H in a hidden-variable model grows asymptotically as*

$$N(H) \propto n^{\frac{3-\tau}{2}(k+B_t(H))+\frac{\tau-1}{2}k_1}, \tag{24}$$

and is thus fully determined by the partition $\mathscr{P}^*$ that optimizes (23).

Proof. The proof of Theorem 4 as well as Lemma 3 follows the same lines as the proof of [43, Theorem 2.1 and Lemma 3.2] for the erased configuration model instead of the hidden-variable model. That proof relies on the fact that the connection probability between vertices with degrees D_i and D_j in the erased configuration model has the same order of magnitude as $\min(D_iD_j/(\mu n), 1)$, which is the same as the connection probability in the hidden-variable model. Thus, the proof of [43, Theorem 2.1] also holds for the hidden-variable model. □

4 Graphlets

We now focus on graphlet counting. Thus, we now only count the number of times the subgraph H appears as an induced subgraph of a hidden-variable model. In particular, edges that are not present in H are also required not to be present in the graph. Because the probability that two edges between vertices of high degree are present equals one (see (5)), this puts a constraint on the number of vertices that typically have high degree. When we again let the degrees in the graphlet scale as n^{α_i}, we see that the probability that an edge (i,j) is present equals one as soon as $\alpha_1+\alpha_j>1$. Thus, for the expected number of graphlets, the optimization problem corresponding to (10) becomes

$$\begin{aligned}&\max(1-\tau)\sum_i \alpha_i + \sum_{(i,j)\in E_H:\alpha_i+\alpha_j<1} \alpha_i+\alpha_j-1\\ &\text{s.t. } \alpha_i+\alpha_j\leq 1 \quad \forall (i,j)\notin E_H,\end{aligned} \tag{25}$$

where E_H denotes the edge set of H. Again, this optimization problem is maximized for $\alpha_i \in 0,\frac{1}{2},1$, so that similarly, the optimization problem corresponding to (18) including the extra constraint then becomes

$$\begin{aligned}B_{g,f}(H) = &\max_{\mathscr{P}}\left[|S_1|-|S_2|-\frac{2E_{S_1}+E_{S_1,S_3}}{\tau-1}\right],\\ &\text{s.t. } (u,v)\in E_H \quad \forall u\in S_2, v\in S_2\cup S_3.\end{aligned} \tag{26}$$

This optimization problem again finds the most likely degrees of vertices that together form the graphlet H. Vertices in S_1 have degrees proportional to a constant, vertices in S_2 have degrees proportional to n and vertices in S_3 have degrees proportional to $\sqrt{n}$. Using this optimization problem, we are also able to find the scaling of the number of graphlets:

Theorem 5 (General graphlets, expected)**.** *Let H be a graphlet on k vertices such that the solution to (26) is unique. The expected number of graphlets $N_g(H)$ in a hidden-variable model grows asymptotically as*

$$\mathbb{E}[N_g(H)] \propto n^{\frac{3-\tau}{2}(k+B_{g,f}(H))}, \tag{27}$$

and is thus fully determined by the partition $\mathscr{P}^*$ that optimizes (26).

For the typical number of graphlets, we obtain a similar result, by adding the constraint $\alpha_i \leq \frac{1}{\tau-1}$ to (25). This results in the optimization problem (like its equivalent version for motifs in (23))

$$\begin{aligned}B_{g,t}(H) = &\max_{\mathscr{P}}\left[|S_1|-|S_2|-\frac{2E_{S_1}+E_{S_1,S_3}+E_{S_1,1}-E_{S_2,1}}{\tau-1}\right],\\ &\text{s.t. } (u,v)\in E_H \quad \forall u\in S_2, v\in S_2\cup S_3.\end{aligned} \tag{28}$$

The corresponding theorem to Theorem 4 for graphlets then becomes:

Theorem 6 (General motifs, typical)**.** *Let H be a graphlet on k vertices such that the solution to (28) is unique and let k_1 denote the number of vertices of degree 1 in H. With high probability, the number of graphlets $N_g(H)$ in a hidden-variable model grows asymptotically as*

$$N_g(H) \propto n^{\frac{3-\tau}{2}(k+B_{g,t}(H))+\frac{\tau-1}{2}k_1}, \tag{29}$$

and is thus fully determined by the partition $\mathscr{P}^*$ that optimizes (28).

Figure 3 shows the typical configuration of all graphlets on 5 vertices, similar to Fig. 1 for all motifs on 5 vertices. Note that the typical configuration of a motif and a graphlet may be substantially different (for example in Fig. 2(b) and its counterpart for motifs).

5 Proof of Theorem 2

Let H be a motif on k vertices such that the dominant motif structure of Theorem 1 contains non-$\sqrt{n}$ vertices. A similar analysis as the triangle example shows that the only motif on 2 vertices is non-self-averaging. Thus, we may assume that $k\geq 3$. Let the expected number of motifs of type H as predicted in Theorem 1 be denoted by $n^{f(H)}$, and the optimal contribution from vertices $1,\dots,k$ in H by $(n^{\alpha_i})_{i\in[k]}$. By Theorem 1,

$$\mathbb{E}[N(H)] \propto \mathbb{E}\left[\#\text{ vertices having weights } (n^{\alpha_i})_{i\in[k]}\right]\mathbb{P}\left(H \text{ present on weights } (n^{\alpha_i})_{i\in[k]}\right) \tag{30}$$

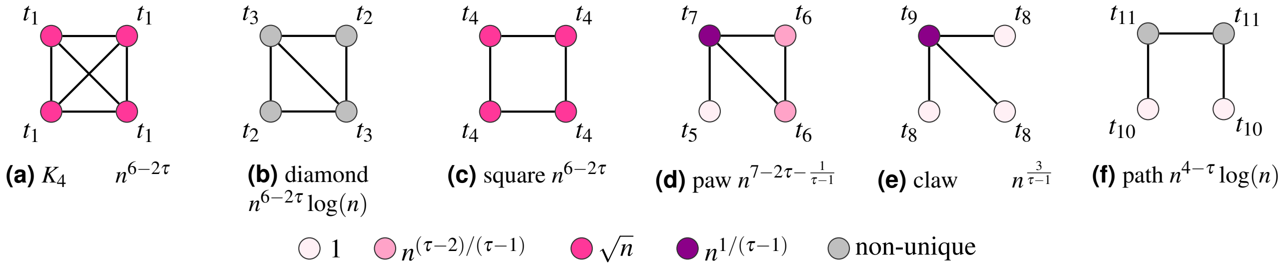

Supplementary Figure 2. Scaling of the typical number of graphlets on 4 vertices in n. The vertex color indicates the typical vertex degree. The vertex labels indicate the vertex types used in Figure 6

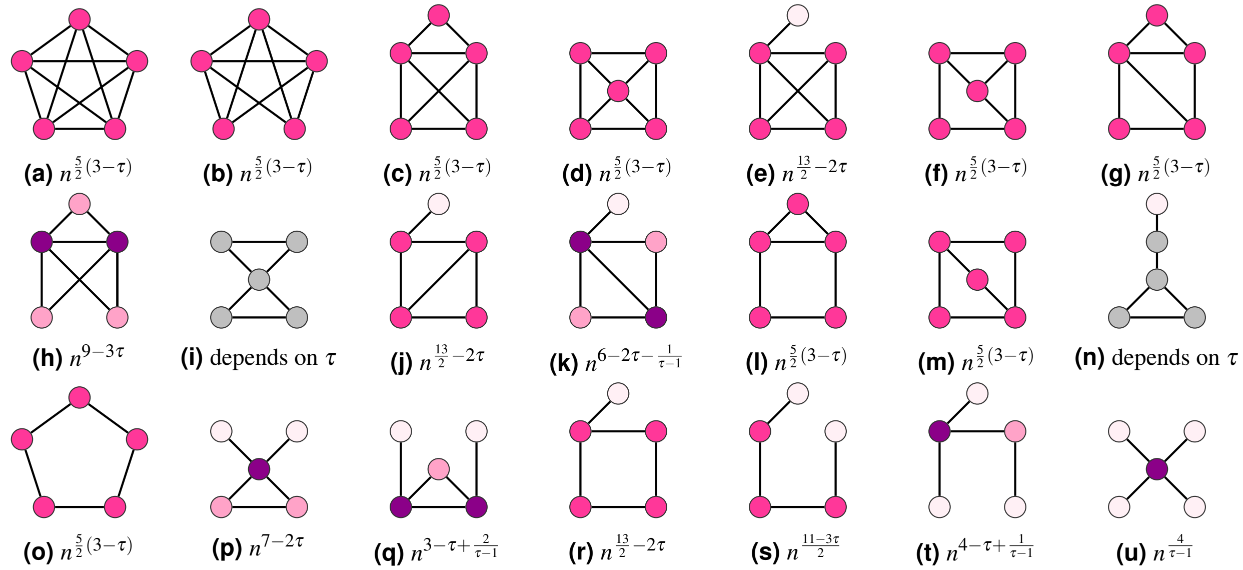

Supplementary Figure 3. Typical number of graphlets for all connected graphs on 5 vertices (constants ignored). The vertex color indicates the dominant vertex degree as in Figure 1.

Suppose there is a vertex v_1 in H with an optimal contribution of weight n vertices, that is $\alpha_1 = 1$. Then, we study the contribution to the variance in (21) from the motif $\tilde{H}$ which is the motif on $2k-1$ vertices where two copies of motif H are merged at their vertex v_1 (see Fig. 4 for an example). We now investigate the expected number of $\tilde{H}$ motifs. In particular, we study the contribution to the expected number of $\tilde{H}$ motifs on vertices with weights of the order of magnitude $(n^{\beta_i})_{i\in[2k-1]}$ with

$$\beta_i = \alpha_{t(i)}, \tag{31}$$

where $t(i)$ is the vertex in H corresponding to vertex i in $\tilde{H}$. Figure 4 gives an example of this contribution when H is a path with 3 vertices. Because of the construction of $\tilde{H}$,

$$\mathbb{P}\left(\tilde{H} \text{ present on weights } (n^{\beta_i})_{i\in[2k-1]}\right) = \mathbb{P}\left(H \text{ present on weights } (n^{\alpha_i})_{i\in[k]}\right)^2. \tag{32}$$

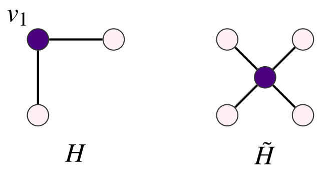

Supplementary Figure 4. Construction of $\tilde{H}$ when H is a path of length 3. $\tilde{H}$ is constructed by merging two copies of H at vertex v_1 with most likely degree n.

Furthermore, the only difference between the vertices in $\tilde{H}$ and two separate versions of H, is that $\tilde{H}$ contains one less vertex of weight proportional to n. The expected number of vertices with weight proportional to n is given by $n^{2-\tau}$ Therefore,

$$\mathbb{E}\left[\#\text{ vertices on weights } (n^{\beta_i})_{i\in[2k-1]}\right] \propto \frac{\mathbb{E}\left[\#\text{ vertices on weights } (n^{\alpha_i})_{i\in[k]}\right]^2}{n^{2-\tau}}. \tag{33}$$

Thus,

$$\begin{aligned}\mathbb{E}[N(\tilde{H})] &\geq \mathbb{P}\left(H \text{ present on weights } (n^{\alpha_i})_{i\in[k]}\right)^2 \frac{\mathbb{E}\left[\#\text{ vertices on weights } (n^{\alpha_i})_{i\in[k]}\right]^2}{n^{2-\tau}} \\ &\propto \mathbb{E}\left[N(H)\right]^2 n^{\tau-2}.\end{aligned} \tag{34}$$

Combining this with (21) of the main text results in

$$\frac{\text{Var}\left(N(H)\right)}{\mathbb{E}\left[N(H)\right]^2} \geq \frac{\mathbb{E}[N(\tilde{H})]}{\mathbb{E}\left[N(H)\right]^2} \geq n^{\tau-2}, \tag{35}$$

which diverges, because $\tau \in (2,3)$. Thus, if the optimal contribution to H satisfies $S_2 \neq \emptyset$, H cannot be self-averaging for $\tau \in (2,3)$.

Now we study the case where H has optimal contribution with $S_1 \neq \emptyset$, but no vertices of weights proportional to n so that $S_2 = \emptyset$. Let $v \in S_1$. The contribution from v to (1) is

$$1 - \frac{2d_{v,S_1} + d_{v,S_3}}{\tau - 1}, \tag{36}$$

where d_{v,S_i} denotes the number of edges from v to vertices in set S_i. Moving v to S_3 would change the contribution to

$$-\frac{d_{v,S_1}}{\tau-1}. \tag{37}$$

Because we know that v in S_1 is the optimal contribution,

$$-\frac{d_{v,S_1}}{\tau-1} < 1 - \frac{2d_{v,S_1} + d_{v,S_3}}{\tau - 1}, \tag{38}$$

or $d_{v,S_1} + d_{v,S_3} \leq \tau - 1$, so that $d_{v,S_1} + d_{v,S_3} \in \{0,1\}$. Thus, every vertex in S_1 has at most 1 edge to other vertices in S_1 or vertices in S_3. Since we have assumed that $S_2 = \emptyset$, and $k > 2$, this means that all vertices in S_1 have degree 1 inside the motif, and are connected to a vertex in S_3. W.l.o.g. assume that v_1 is a vertex such that $v_1 \in S_3$ and v_1 has at least one connection to a vertex in S_1. As in the previous proof, we consider $\tilde{H}$ constructed by merging two copies of H at v_1, as illustrated in Figure 5. Define $(\alpha_i)_{i\in[k]}$ as the maximal contribution to H. We define

$$\beta_i = \begin{cases}\alpha_{t(i)} & t(i) \neq 1, \\ 1 & t(i) = 1.\end{cases} \tag{39}$$

That is, we study the contribution where all vertices in $\tilde{H}$ except v_1 have the same weight as in their counterpart in H. The weight of v_1 is proportional to n, whereas the counterpart of v_1 in H had weight proportional to $\sqrt{n}$, as in the illustration in Fig. 5. We again study the contribution to $\mathbb{E}\left[N(\tilde{H})\right]$ from vertices of weights $(n^{\beta_i})_{i\in[2k-1]}$. We now compare the probability that $\tilde{H}$ exists on vertices of weights $(n^{\beta_i})_{i\in[2k-1]}$ to the probability that two copies of H exist on weights $(n^{\alpha_i})_{i\in[k]}$. The difference between these two probabilities is that vertex v_1 in $\tilde{H}$ has weight n instead of $\sqrt{n}$ in H. In H, v_1 is connected to at least one vertex of weight proportional to 1. The probability of this connection to be present is proportional to $n^{-1/2}$. In $\tilde{H}$, v_1 has weight n, so that the probability that the corresponding connections occur in $\tilde{H}$ is proportional to 1. The connection probabilities on vertices not connected to v_1 do not change, so that

$$\mathbb{P}\left(\tilde{H} \text{ present on weights } (n^{\beta_i})_{i\in[2k-1]}\right) \geq \frac{\mathbb{P}\left(H \text{ present on weights } (n^{\alpha_i})_{i\in[k]}\right)^2}{n^{-1}}. \tag{40}$$

Since the difference between the vertices of two copies of H and $\tilde{H}$ is that we remove two vertices of weight $\sqrt{n}$ and add one vertex of weight proportional to n, we obtain

$$\mathbb{E}\left[\#\text{ vertices on weights } (n^{\beta_i})_{i\in[2k-1]}\right] \propto \frac{\mathbb{E}\left[\#\text{ vertices on weights } (n^{\alpha_i})_{i\in[k]}\right]^2 n^{2-\tau}}{n^{3-\tau}}, \tag{41}$$

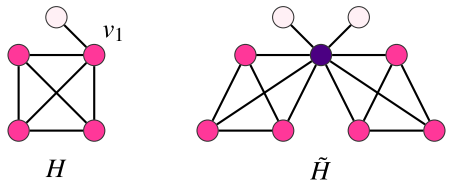

Supplementary Figure 5. Example of the construction of $\tilde{H}$. $\tilde{H}$ is constructed by merging two copies of H at vertex v_1: a vertex with most likely degree $\sqrt{n}$ that is connected to a vertex of degree 1.

where we have used that the number of vertices with degree proportional to $\sqrt{n}$ is $n^{(3-\tau)/2}$ by (8) and the number of vertices with degree proportional to n scales as $n^{2-\tau}$. This results in

$$\mathbb{E}[N(\tilde{H})] \geq \mathbb{P}\left(H \text{ present on degrees } (n^{\alpha_i})_{i\in[k]}\right)^2 \geq \mathbb{E}\left[\# \text{ vertices on degrees } (n^{\alpha_i})_{i\in[k]}\right]^2 \\ \propto \mathbb{E}\left[N(H)\right]^2 \tag{42}$$

so that by (21)

$$\frac{\mathrm{Var}\left(N(H)\right)}{\mathbb{E}\left[N(H)\right]^2} \geq \frac{\mathbb{E}[N(\tilde{H})]}{\mathbb{E}\left[N(H)\right]^2} \propto 1, \tag{43}$$

which does not converge to zero, so that the motif is not self-averaging.

Graphlet fluctuations

The fluctuations of the number of graphlets can be studied similarly as the motif fluctuations. Again, (21) holds, but now it only includes graphlets $H_1,\dots,H_l$ that can be constructed by merging two copies of H at one or more vertices, with the additional constraint that after merging the copies of H, both copies still form induced subgraphs of H. As an example, consider 2-path fluctuations. The subgraphs that can be constructed from merging two 2-paths are the subgraphs in Fig. 3s, 3t, 3u, 2c, 2d, 2e, 2f, Fig. 3a and 3b. However, the merged subgraph of Fig. 2d and Fig. 2b does not contain two induced copies of graphlets, and therefore these subgraphs are excluded from equation (21). Other than that, the procedure to determine for any graphlet whether it is self-averaging or not is the same as the procedure for motifs, using Theorem 5 to find the expected order of magnitude of the number of merged graphlets.

6 Optimal diamond motif

We now study the optimizers of (6) for the diamond motif (Fig. 3b) for all $\tau \in (2,3)$. Let i and j be the vertices at the diagonal of the diamond, and k and s the corner vertices. By [49], (6) is maximized by $S_3 = \{i,j,k,s\}$ or $S_1 = \{k,s\}$ and $S_2 = \{i,j\}$ and by Lemma 2 these are two optimizers of (6), yielding a contribution of $2(1-\tau)$.

By the argument in Lemma 2, for any vertex u, the optimizer of (6) is either given by $\alpha_u = 0$ or constrained by $\alpha_u + \alpha_v = 1$ for at least one edge $(u,v) \in E_H$. The above two optimizers show that for all vertices in the diamond motif, there is at least one such constrained edge. Therefore, $\alpha_i + \alpha_j + \alpha_k + \alpha_s = 2$ must hold.

Then, to find all optimizers, we solve

$$(1-\tau)(\alpha_i+\alpha_j+\alpha_k+\alpha_s) + \mathbb{1}_{\{\alpha_i+\alpha_j<1\}}(\alpha_i+\alpha_j-1) + \mathbb{1}_{\{\alpha_i+\alpha_k<1\}}(\alpha_i+\alpha_k-1) + \mathbb{1}_{\{\alpha_i+\alpha_s<1\}}(\alpha_i+\alpha_s-1) \\ + \mathbb{1}_{\{\alpha_j+\alpha_s<1\}}(\alpha_j+\alpha_s-1) + \mathbb{1}_{\{\alpha_j+\alpha_k<1\}}(\alpha_j+\alpha_k-1) = 2(1-\tau). \tag{44}$$

Using that $\alpha_i+\alpha_j+\alpha_k+\alpha_s = 2$ this reduces to

$$\alpha_i+\alpha_j+\alpha_k+\alpha_s = 2, \quad \alpha_i+\alpha_j \geq 1, \quad \alpha_i+\alpha_k \geq 1, \quad \alpha_i+\alpha_s \geq 1, \quad \alpha_j+\alpha_s \geq 1, \quad \alpha_j+\alpha_k \geq 1. \tag{45}$$

The constraints $\alpha_i+\alpha_k \geq 1$, $\alpha_j+\alpha_s \geq 1$ together with $\alpha_i+\alpha_j+\alpha_k+\alpha_s = 2$ yield $\alpha_i+\alpha_k = 1$ and $\alpha_j+\alpha_s = 1$. Similarly, $\alpha_i+\alpha_s = 1$ and $\alpha_k+\alpha_j = 1$, resulting in $\alpha_i = \alpha_j =: \beta$ and $\alpha_k = \alpha_s = 1-\beta$. Furthermore, the constraint $\alpha_i+\alpha_j \geq 1$ yields $\beta \in [1/2,1]$.

7 Graphlet counts

	**Gowalla**	**Oregon**	**Enron**	**PGP**	**HEP**
**claw**	$5.96 \cdot 10^{14}$	$3.13 \cdot 10^{14}$	$4.48 \cdot 10^{14}$	$4.04 \cdot 10^{6}$	$1.23 \cdot 10^{6}$
**path**	$1.52 \cdot 10^{14}$	$7.03 \cdot 10^{7}$	$1.37 \cdot 10^{14}$	$2.72 \cdot 10^{6}$	$2.12 \cdot 10^{6}$
**paw**	$3.11 \cdot 10^{14}$	$2.01 \cdot 10^{7}$	$3.76 \cdot 10^{8}$	$1.96 \cdot 10^{6}$	$5.31 \cdot 10^{5}$
**diamond**	$8.60 \cdot 10^{7}$	$9.61 \cdot 10^{5}$	$2.25 \cdot 10^{7}$	$2.74 \cdot 10^{5}$	$3.55 \cdot 10^{4}$
**square**	$4.24 \cdot 10^{7}$	$7.46 \cdot 10^{4}$	$6.76 \cdot 10^{6}$	$2.16 \cdot 10^{4}$	$6.84 \cdot 10^{3}$
**K4**	$6.09 \cdot 10^{6}$	$3.05 \cdot 10^{4}$	$2.34 \cdot 10^{6}$	$2.39 \cdot 10^{5}$	$6.56 \cdot 10^{4}$

Supplementary Table 1. Graphlet counts in the five data sets.
